# ﻿A phylogenetic and morphological study of the *Tectariafuscipes* group (Tectariaceae), with description of a new species

**DOI:** 10.3897/phytokeys.195.80452

**Published:** 2022-05-09

**Authors:** Shi-Yong Dong, Shu-Hang Li, Ling Huang, Shi-Shi Tan, Zheng-Yu Zuo

**Affiliations:** 1 Key Laboratory of Plant Resources Conservation and Sustainable Utilization, South China Botanical Garden, Chinese Academy of Sciences, Guangzhou 510650, China South China Botanical Garden, Chinese Academy of Sciences Guangzhou China; 2 Center of Conservation Biology, Core Botanical Gardens, Chinese Academy of Sciences, Guangzhou 510650, China Core Botanical Gardens, Chinese Academy of Sciences Guangzhou China; 3 University of Chinese Academy of Science, Beijing, 100049, China University of Chinese Academy of Science Beijing China; 4 Germplasm Bank of Wild Species, Kunming Institute of Botany, Chinese Academy of Sciences, Kunming 650201, China Kunming Institute of Botany, Chinese Academy of Sciences Kunming China; 5 School of Life Sciences, Yunnan University, Kunming 650500, China Yunnan University Kunming China

**Keywords:** fern, mainland Asia, molecular phylogeny, morphology, taxonomy

## Abstract

The fern species *Tectariafuscipes* and morphologically similar species, which are common in tropical and subtropical mainland Asia, constitute a taxonomically confusing group. To better understand species boundaries and relationships within the *T.fuscipes* group, we conducted phylogenetic analyses of five plastid regions and morphological observations of herbarium specimens and living plants. As a result, we produced a generally well-resolved phylogeny of the *T.fuscipes* group and related species in Asia. The phylogenetic analyses supported the monophyly of the *T.fuscipes* group, which includes *T.dissecta*, *T.fuscipes*, *T.ingens*, *T.paradoxa*, *T.setulosa*, *T.subfuscipes*, *T.subsageniacea* and a new species, but excludes *T.kusukusensis*. However, *T.fuscipes*, *T.subfuscipes* and *T.subsageniacea* are almost indistinguishable in morphology, which form a complex characterised by the black linear-lanceolate stipe scales. The new species found in southern China and Vietnam is described here as *T.fungii*. It is similar to the *T.fuscipes* complex and *T.kusukusensis*, but differs from the former mainly by its brown-castaneous lanceolate stipe scales and from the latter by having nearly hairless laminae (versus frond axes abaxially bearing copious hairs).

## ﻿Introduction

*Tectaria* Cav. is a pantropical and south-temperate fern genus of about 264 species (Hassler 2004–2022). It belongs to Tectariaceae of eupolypods I in the classification of PPG I (2016). *Tectaria* in the modern sense is based on molecular phylogenetic data (Ding et al. 2014; Wang et al. 2014; Chen et al. 2018), covering *Aenigmopteris* Holttum, *Cionidium*T. Moore, *Ctenitopsis* Ching ex Tardieu & C. Chr., *Heterogonium* C. Presl, *Psomiocarpa* C. Presl, *Tectaridium* Copel. and other satellite genera previously recognised. The species now included in *Tectaria* are morphologically very diverse, which makes *Tectaria* as a genus difficult to be distinguished from its allied genera *Hypoderris* R. Br. ex Hook. and *Triplophyllum* Holttum (Moran et al. 2014). For the majority of *Tectaria*, the diagnostic features include the rhizome and stipe covered with lanceolate scales, the less dissected and herbaceous fronds with their axes adaxially non-grooved and bearing ctenitoid hairs, anastomosing veins and discrete round sori on the abaxial surface of laminae (Ching 1931). According to recent studies (Ding et al. 2014; Zhang et al. 2017; Dong et al. 2018a), *Tectaria* phylogenetically includes four major clades, with one clade confined to the Neotropics and the other three in the Old World; but none of them was found having a synapomorphic morphology.

In tropical and subtropical Asia, *Tectariafuscipes* (Wall. ex Bedd.) C. Chr. and morphologically similar species constitute a taxonomically confusing group. They are characterised by the fronds with basal pinnae basiscopically produced and veins being wholly free or, as in *T.fuscipes*, with veins anastomosing to form costal areoles in its sterile fronds (Holttum 1991). In the area from Taiwan Island westwards to South Asia, the species having the frond shape like that of *T.fuscipes* and free veins include *T.dissecta* (G. Forst.) Lellinger, *T.fuscipes*, *T.ingens* (Atk. ex C.B. Clarke) Holttum, *T.kusukusensis* (Hayata) Lellinger, *T.setulosa* (Baker) Holttum, *T.paradoxa* (Fée) Sledge, *T.subfuscipes* (Tagawa) C.M. Kuo and *T.subsageniacea* (Christ) Christenh. (Holttum 1988; Kuo 1997; Zink 2006; Lindsay and Middleton 2012 onwards; Xing et al. 2013; Fraser-Jenkins et al. 2018), as well as an unidentified taxon which was labelled as “*Tectaria* sp.1” by Dong et al. (2018a). Of these species, the most contentious species are *T.subfuscipes* and *T.subsageniacea*. *Tectariasubfuscipes* was described for the plants from Taiwan Island that are morphologically similar to *T.fuscipes*, but different in the free veins and monomorphic fronds (Tagawa 1939). When revising *Tectaria* species with free or partly anastomosing veins in Asia, Holttum (1988) treated *T.subfuscipes* as a synonym of *T.fuscipes*. However, both *T.subfuscipes* and *T.fuscipes* were accepted as distinct species in the fern flora of Taiwan (Kuo 1997; Knapp 2014). In southern China, *Aspidiumsubsageniaceum*Christ (1906) (= *T.subsageniacea*), *Ctenitopsisglabra* Ching & Chu H. Wang (Ching and Wang 1964) and *Ctenitopsisacrocarpa* Ching (Ching and Wang 1981) were proposed as morphologically similar species to *T.fuscipes*. The first author (Dong) agreed with a broad concept of *T.fuscipes* sensu Holttum (1988) and treated all these names for plants from southern China as synonyms of *T.fuscipes* (Dong et al. 2002; Dong 2017). However, the phylogenetic analyses by Zhang et al. (2017) showed that *T.subsageniacea* was in a different subclade from *T.fuscipes*. The relationships between *T.fuscipes*, *T.subfuscipes* and *T.subsageniacea* had not been resolved in Zhang et al. (2017).

To better resolve the relationships between species and explore species boundaries within the *T.fuscipes* group, we conducted phylogenetic analyses of plastid sequences with an enlarged sampling and made morphological observations of herbarium specimens and living plants. Specifically, the purposes of this study are to construct a phylogeny of the *T.fuscipes* group and related species in mainland Asia, to detect morphological differences amongst *T.fuscipes*, *T.subfuscipes* and *T.subsageniacea* and to determine the identity of the “*Tectaria* sp.1” in Dong et al. (2018a).

## ﻿Methods

For morphological comparisons, the first author (Dong) studied in person the specimens of *Tectaria* in these Herbaria: BM, BO, DACB, E, GAUA, HITBC, HN, HNU, IBK, IBSC, K, KUN, L, LAE, P, PE, PNH, PYU, SING and TAIF. In addition, we conducted morphological observations of living plants in the wild of Bangladesh, China and Vietnam. For both herbaria specimens and living plants, we focused on the states and the variation of stipe scales (shape and colour), lamina hairs, fronds dimorphism, venation and sori distribution, which were characters frequently used by previous authors (e.g. Holttum 1988; Shieh 1994; Kuo 1997; Wang 1999; Xing et al. 2013; Fraser-Jenkins et al. 2018) to recognise *T.fuscipes* and morphologically similar species.

The sampling for phylogenetic analyses in this study focused on species with free or relatively simple anastomosing species in the Old World which constitute one of four major clades in *Tectaria* (Ding et al. 2014; Zhang et al. 2017; Dong et al. 2018a). A total of 51 specimens were analysed, including three specimens of the unidentified taxon, 16 of *Tectariafuscipes* s. l. (including *T.subfuscipes* and *T.subsageniacea*) and representatives of all known species with free veins in mainland Asia and adjacent islands, except for *T.hennipmanii* (Tagawa & K. Iwats.) S.Y. Dong, a very rare species and hitherto represented only by its type from Thailand. Of the 51 specimens, 23 were newly sequenced and analysed in this study (Appendix 1). The same five plastid regions (*atpB*, *ndhF* plus *ndhF-trnN*, *rbcL*, *rps16-matK* plus *matK* and *trnL-F*) used in previous studies (Ding et al. 2014; Dong et al. 2018a) were followed here to infer the phylogeny.

Genomic DNA of the 23 newly-added samples were extracted from silica-dried leaves, except for that of *T.paradoxa*, for which we instead used leaf fragments of herbarium specimens. The subsequent amplifications were carried out with the primers described in Ding et al. (2014) according to the standard protocols of PCR. The PCR products were sequenced using the BigDye Terminator Cycle Sequencing kit according to the manufacturer’s instructions (Applied Biosystems, Foster City, CA, USA) on an ABI 3730XL automated sequencer. Newly-generated sequences and those from GenBank were aligned individually using MAFFT (Katoh et al. 2005) and subsequently adjusted manually in BioEdit version 7.2.0 (Hall 1999). We then concatenated the five regions of each sample into a combined matrix for the following analyses.

We analysed the matrix using Bayesian Inference (BI), Maximum Likelihood (ML) and Maximum Parsimony (MP), respectively. The software jModeltest2 (Posada 2008) was used to determine the best-fitting substitution models for the combined sequences and the results suggested GTR+G+I as the best-fitting model in both BI and ML analyses. The BI analysis was conducted with MrBayes 3.2.6 (Ronquist et al. 2012), using 10 million generations with one tree sampled every 1,000 generations; four runs with four chains were performed in parallel. The first 25% trees were discarded as burn-in. The ML analysis was conducted using raxmlGUI2.0 (Edler et al. 2020). A thorough tree search for the best ML tree was performed. The ML Bootstrap analysis was performed with 1000 replications; bipartition information from the bootstrap trees was drawn on the best ML tree. The MP analysis was conducted in PAUP* version 4.0d100 (Swofford 2002), with all characters weighted equally and gaps considered as missing data. One thousand heuristic replicated searches were carried out using random stepwise addition with branch swapping by tree bisection-reconnection (TBR), saving 100 trees per replicate. Bootstrap values (BS) were calculated with 1000 heuristic bootstrap replicates, one random sequence addition and TBR swapping.

## ﻿Results

### ﻿Scales

**Figure 1.**
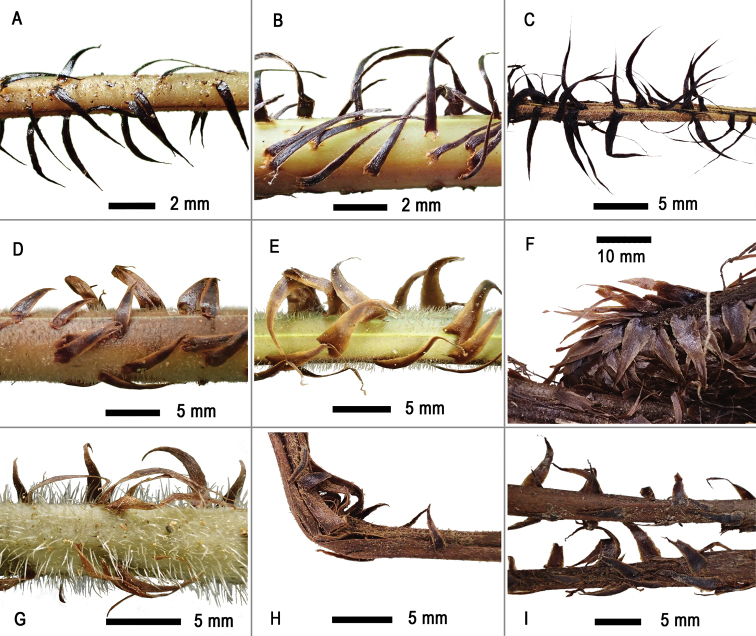
Comparison of stipe scales in *Tectariafuscipes* and morphologically similar species **A***T.fuscipes* (*Dong 5194*, IBSC) **B***T.subsageniacea* (*Dong 4270*, IBSC) **C***T.subfuscipes* (*Chang 20140503021*, TAIF) **D***T.* sp. (*Dong 5096*, IBSC) **E***T.setulosa* (*Dong 4782*, IBSC) **F***T.ingens* (*Miehe et al. 13093007*, SING) **G***T.kusukusensis* (*Dong 4851*, IBSC) **H***T.paradoxa* (*Fraser-Jenkins FN77*, TAIF) **I***T.dissecta* (*Chang 20160125*, TAIF).

For the *Tectariafuscipes* group, the colour of stipe scales can be determined as two basic states: brown and black. Scales in *T.fuscipes*, *T.subfuscipes* and *T.subsageniacea* are constantly black, with or without very narrow brown margins, whereas, in the unidentified taxon and other species, they are brown, sometimes brown-castaneous. The black scales are associated with a linear-lanceolate shape, which measures 0.5–1 mm wide (Fig. 1A–C). In contrast, the brown scales are generally broader, usually 1–1.5 mm or, as in *T.ingens* and *T.setulosa*, up to 3 mm wide (Fig. 1D–I).

### ﻿Lamina hairs

Based on the abundance of hairs on the abaxial surface of costae, which are easily observable in herbarium specimens, the fronds can be generally recognised as either nearly hairless or obviously hairy for *T.fuscipes* and morphologically similar species. We observed fronds with dense hairs in *T.kusukusensis*, some collections of *T.fuscipes* from Taiwan and Bangladesh and some collections of *T.ingens* and *T.setulosa*. In the unidentified taxon and other species of the *T.fuscipes* group, the fronds are nearly hairless. The abundance of lamina hairs is variable in *T.fuscipes*, *T.ingens* and *T.setulosa*. We noticed that the fronds can be hairless or hairy even in a single population of *T.fuscipes*, such as *Lu 16213* (TAIF) from Bangladesh.

### ﻿Frond dimorphism

**Figure 2.**
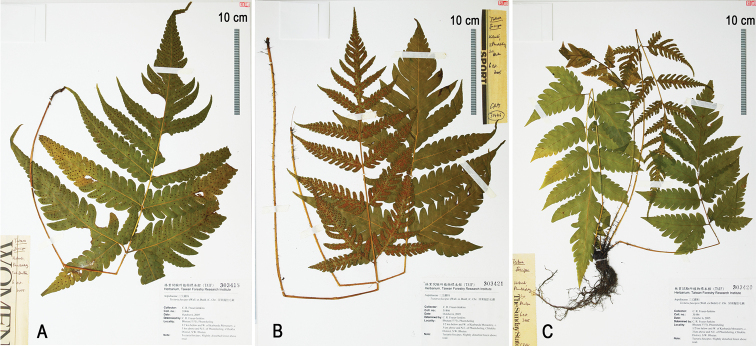
Three states of fronds’ fertile-sterile dimorphism in one population of *Tectariafuscipes* from Bhutan (*Fraser-Jenkins 31446*, TAIF) **A** monomorphic **B** subdimorphic **C** dimorphic.

The fronds of all species in the *T.fuscipes* group are more or less dimorphic, i.e. a fertile lamina being contracted to a certain extent as compared with a sterile lamina in a population. Our observations showed that the obvious dimorphism of fronds is frequent in *T.fuscipes*, sometimes occurs in *T.subsageniacea*, but is scarce in other species of the *T.fuscipes* group including the unidentified taxon. However, it is difficult to determine the fronds as monomorphic or dimorphic for *T.fuscipes* and *T.subsageniacea* because the variation from monomorphic to dimorphic is gradual and continuous. We detected different variation tendencies of frond dimorphism instead of clear differences in these two taxa. Namely, the fronds tend to be dimorphic in *T.fuscipes*, but are mostly nearly monomorphic in *T.subsageniacea*. Notably, we observed three different states in a single collection of *T.fuscipes* from Bhutan (*Fraser-Jenkins 31446*, TAIF) (Fig. 2), which indicates the instability of frond dimorphism in this species.

### ﻿Venation

**Figure 3.**
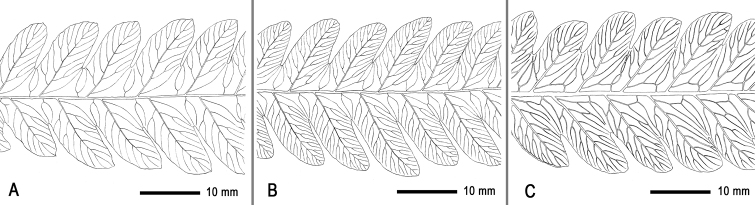
Three states of venation in the *Tectariafuscipes* group **A** free as in *T.subsageniacea* (*Dong 3856*, IBSC) **B** intermediate (veins unstably forming areoles along costae) as in *T.subsageniacea* (*Dong 4585*, IBSC) **C** costal-vein-anastomosing (veins regularly forming areoles along costae) in *T.fuscipes* (*Dong 4686*, IBSC).

The venation in the *T.fuscipes* group can be recognised as three states: free (Fig. 3A), intermediate (Fig. 3B) and costal-veins-anastomosing (i.e. veins along costae regularly forming costal areoles) (Fig. 3C). The costal-vein-anastomosing venation was observed only in *T.fuscipes*; the intermediate venation was found in *T.fuscipes*, *T.subfuscipes* and *T.subsageniacea*; and the free venation was found in the unidentified taxon and all species of the *T.fuscipes* group. The intermediate venation covers a variation range, being free on some pinnae of a frond, but forming several costal areoles (continuous or not) on other pinnae of the same frond. The intermediate venation occurs frequently in *T.subfuscipes* from Taiwan Island, *T.subsageniacea* in southern China and Indochina and *T.fuscipes* in north-eastern India and nearby regions of East Himalayas. Our statistics showed that, in East Himalayas, there are about 54.7% of herbarium specimens of *T.fuscipes* having the free or intermediate venation; for *T.subfuscipes* from Taiwan Island, there are about 15% of herbarium specimens having the intermediate venation. Notably, we found that the free venation and the costal-vein-anastomosing venation can simultaneously occur in a single collection from north-eastern India (e.g. *Fraser-Jenkins FN57*, *174*, *31446*, all in TAIF).

### ﻿Sori arrangement

**Figure 4.**
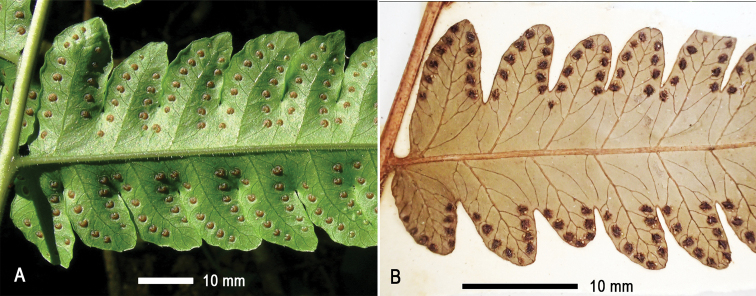
Two states of sori arrangement in the *Tectariafuscipes* group **A** sori medial, borne on segments almost from base to tip as in *T.* sp. (*Dong 1589*, IBSC) **B** sori supramedial, borne on distal half of segments in *T.paradoxa* (*Thwaites 3061*, BO, TUB).

Sori are regularly arranged in two rows on the ultimate segments of pinnae in the *T.fuscipes* group. They are medial (positioned between mid-rib and margin) and are distributed nearly from base to tip on ultimate segments (Fig. 4A) in all species of this group, except *T.paradoxa*; in the latter, sori are mostly supramedial and restricted to the middle and apex of segments (Fig. 4B).

### ﻿Molecular phylogeny

The concatenated alignment of the five plastid regions (*atpB*, *ndhF* plus *ndhF-trnN*, *rbcL*, *rps16-matK* plus *matK* and *trnL-F*) accounts for 5865 base pairs, including 64 indels. Of the total 5865 characters, 1027 are variable and 688 are parsimony informative. The length of the best MP trees is 1763 (consistency index = 0.633, retention index = 0.800). The likelihood score of the ML tree is -18708.288.

**Figure 5.**
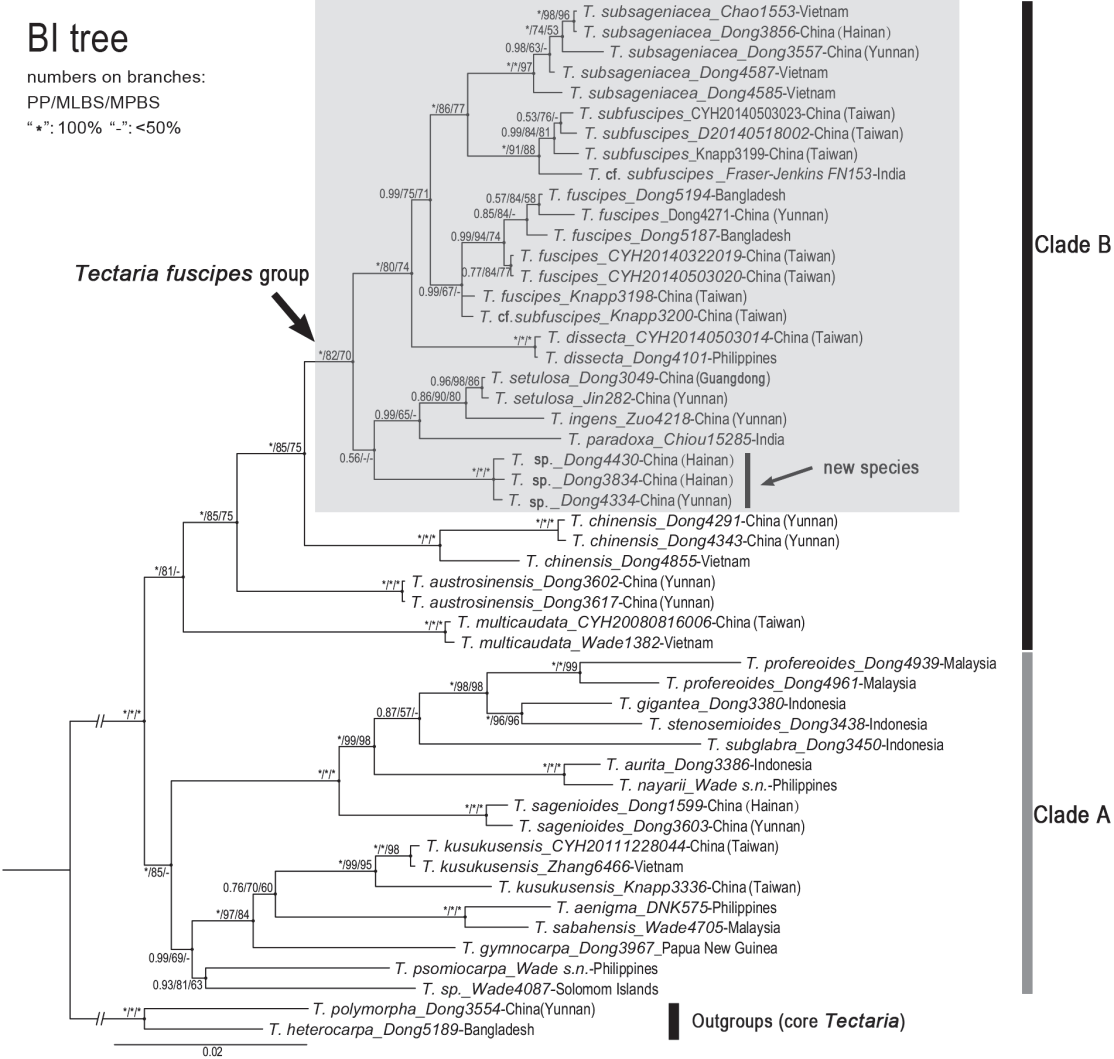
Bayesian consensus tree of the *Tectariafuscipes* group and allied species with free or relatively simply anastomosing veins in the Old World, based on concatenated plastid regions of *atpB, ndhF* plus *ndhF-trnN, rbcL, rps16-matK* plus *matK* and *trnL-F*.

The topology resulted from the BI analysis is consistent with that of the ML analysis, while in the tree from the MP analysis, the samples are not so well resolved as in the BI or ML tree. There are no obvious conflicts between the topology of BI (or ML) analysis and that of MP analysis, except for the position of *T.subglabra* (Holttum) S.Y. Dong, which was resolved as sister to *T.aurita* (Sw.) S. Chandra and *T.nayarii* Mazumdar in the MP tree with low support (MPBS = 68%), while as sister to *T.profereoides* (Christ) S.Y. Dong and allied species in the BI or ML tree with poor support (PP = 87%, MLBS = 57%). There is no conflict involving the position of the *T.fuscipes* group and species relationships within this group between the trees inferred from different analyses. Therefore, we adopt the topology resulting from the BI analysis (Fig. 5), where most samples were well resolved and 75% nodes received strong support (PP = 1.0 or 0.99, MLBS > 80%), as a base to describe and discuss the relationships involving the *T.fuscipes* group.

All in-group samples were resolved into two large clades and each clade generally corresponds to a geographical region. The samples from Malesia clustered into a clade (Clade A) and those from mainland Asia and adjacent islands (except for *T.kusukusensis* and *T.sagenioides* (Mett.) Christenh.) clustered into another clade (Clade B) (Fig. 5). The *T.fuscipes* group was resolved as a monophyletic clade, forming a sister relationship with *T.chinensis* (Ching & Chu H. Wang) Christenh. Within the *T.fuscipes* group, all samples were further resolved into three clades: (1) the unidentified taxon represented by three specimens, (2) *T.paradoxa*, *T.ingens* and *T.setulosa* and (3) *T.dissecta*, *T.fuscipes*, *T.subfuscipes* and *T.subsageniacea*. The unidentified taxon was resolved as sister to the *T.paradoxa* clade with poor support values.

The 16 specimens representing *Tectariafuscipes* s. l. (including *T.subfuscipes* and *T.subsageniacea*) were well resolved into three clades. All specimens of *T.fuscipes* s. str., including one (*Knapp 3200*) with a morphology similar to *T.subfuscipes*, clustered together, forming a sister clade to the rest. All specimens of *T.subsageniacea* from mainland China and Vietnam were well resolved in a clade, forming a sister relationship with the clade containing three specimens of *T.subfuscipes* from Taiwan Island and one *T.subfuscipes*-like specimen from India.

## ﻿Discussion

### ﻿A new species supported by molecular and morphological evidence

The results of our morphological comparisons and phylogenetic analyses of plastid sequences support an undescribed species in the *Tectariafuscipes* group. As shown in the phylogenetic tree (Fig. 5), three specimens of the undescribed species (*Dong 3834*, *4334*, *4430*) formed a relatively independent, well-supported subclade in Clade B. Its herbarium specimens were frequently misidentified as *T.fuscipes* sensu Holttum (1988) or *T.kusukusensis* (Ching 1938 as *Ctenitopsiskusukusensis* (Hayata) Ching). The new species differs from *T.fuscipes* s. l. (including *T.subfuscipes* and *T.subsageniacea*) mainly in the brown or castaneous and relatively broad stipe scales (Fig. 1D) (versus black and narrow-lanceolate, Fig. 1A) and differs from *T.kusukusensis* in its frond-axes (stipe, rachis and costae) bearing sparse and short hairs (ca. 0.5 mm) (versus dense and in 1–1.5 mm long, Fig. 1G). Additionally, the stipe scales appear somewhat different in the two species, being slightly lustrous and brown or castaneous in the new species (Fig. 1D), but dull brown in *T.kusukusensis* (Fig. 1G). The new species is formally described as *T.fungii* in the taxonomic treatment below.

### ﻿Circumscription and interspecific relationships of the *Tectariafuscipes* group

The morphological and phylogenetic analyses support the close affinity amongst *T.dissecta*, *T.fungii*, *T.fuscipes*, *T.ingens*, *T.paradoxa*, *T.setulosa*, *T.subfuscipes* and *T.subsageniacea*. These taxa constitute a natural group, namely *T.fuscipes* group (Fig. 5), which is morphologically characterised by the free venation (or sometimes costal-vein-anastomosing venation), fronds with proximal pinnae basiscopically divided with segment or pinnules elongated and sori being terminal on free veins and in two rows on ultimate segments. The most closely allied species to the *T.fuscipes* group is indicated to be *T.chinensis*, which differs in its high number of anastomosing veins (having costal areoles and additional areoles) and sori on anastomosed veins or non-apical on free veins (Dong et al. 2018b).

*Tectariakusukusensis* has the characteristic morphology of the *T.fuscipes* group, but should not be considered as a member of this group. *Tectariakusukusensis* agrees well with the free-veined *T.fuscipes* and *T.dissecta* in lamina dissection, the shape of basal pinnae, venation and sori arrangement, but differs in having copious hairs on fronds (Shieh 1994; Kuo 1997; Xing et al. 2013). It is distributed in tropical East Asia (Holttum 1988), falling within the distribution range of *T.fuscipes*. However, the phylogenetic analyses in this and previous studies (Zhang et al. 2017; Dong et al. 2018a) consistently resolved *T.kusukusensis* in a different clade from the *T.fuscipes* group. Its close relatives are suggested to be *T.aenigma* (Copel.) C.W. Chen & C.J. Rothf., *T.sabahensis* C.W. Chen & C.J. Rothf. and *T.gymnocarpa* Copel. (Fig. 5), which are all confined to Malesia. It is likely that *T.kusukusensis* had originally been derived in Malesia and later colonised in tropical East Asia.

Within the *T.fuscipes* group, *T.fuscipes*, *T.subfuscipes* and *T.subsageniacea* constitute a closely allied subgroup, which is supported by the black linear-lanceolate stipe scales. The closely allied species to them is suggested to be *T.dissecta*, which differs in its broad lanceolate stipe scales and much narrow fronds and is the only species in the *T.fuscipes* group mainly distributed in Malesia to the Pacific Islands (Holttum 1991). The remaining four species, *T.fungii*, *T.ingens*, *T.paradoxa* and *T.setulosa*, constitute another subgroup in the *T.fuscipes* group. Of the four, *T.ingens* and *T.setulosa* are closely allied to each other. The relationships amongst *T.ingens* plus *T.setulosa*, *T.paradoxa* and the new species *T.fungii* currently remain uncertain, which is probably due to the incomplete sequences of the only representative of *T.paradoxa* (*Chiou 15285*) analysed in this study. As shown in the tree produced by Dong et al. (2018a), where *T.paradoxa* was not sampled and complete sequences are available for all accessions in the *T.fuscipes* group, *T.fungii* (then named *Tectaria* sp.1) was well resolved as sister to *T.setulosa*. Therefore, it is expected that the relationships between the new species and other species will be well resolved when better DNA materials are available for *T.paradoxa*.

### ﻿Indistinct morphology between *Tectariafuscipes*, *T.subfuscipes* and *T.subsageniacea*

According to the current sampling, the phylogenetic analyses supported *T.fuscipes*, *T.subfuscipes* and *T.subsageniacea* as three different lineages (Fig. 5). However, we currently did not find a morphological character which can clearly distinguish one from the other two species. Especially for specimens from Taiwan Island and East Himalayas, they are difficult to be determined as either *T.fuscipes* or *T.subfuscipes*. *Tectariasubfuscipes* was regarded as distinguishable from *T.fuscipes* by the absence of costal areoles and the nearly monomorphic (versus dimorphic) fronds (Kuo 1997). In fact, our examinations showed that the free or anastomosing venation cannot be used to group specimens from East Himalayas into different taxa because those two states of venation were observed occurring in a single collection there. Similarly, we found that about 15% of specimens from Taiwan Island cannot be identified as *T.fuscipes* or *T.subfuscipes*, based on the variation of venation. It is neither feasible to recognise the three species by the variation of frond dimorphism (monomorphic or dimorphic), because the fertile fronds are contracted to different extents as compared with the sterile fronds in all these species nor is it possible to draw a line between the two states of fronds. In our opinion, *T.fuscipes*, *T.subfuscipes* and *T.subsageniacea* constitute a species complex which are currently indistinguishable in morphology.

We noticed three collections from the same locality (a forest valley in Gaoxiong, Taiwan Island), i.e. *Knapp 3198*, *3199*, *3200*, which exhibit gradually varied states of frond dimorphism and venation, but were resolved into two clades in the phylogenetic tree (Fig. 5). This result and the comparatively large size of *Knapp 3200* suggest possible hybridisations existed between *T.fuscipes* and *T.subfuscipes*. Unfortunately, we hitherto have very few cytological or reproductive data for these species. Only one specimen (*Kato et al. 2624*) from southern Yunnan was reported by Kato et al. (1992) having 80 (2*x*) somatic chromosome numbers and sexual reproduction. One specimen (*Dong 3557*) also from southern Yunnan was examined having the same number (2*n* = 80) (unpubl. data). To better understand the morphological variations amongst *T.fuscipes*, *T.subfuscipes* and *T.subsageniacea*, more cytological and reproductive data, as well as more sampling in phylogenetic analyses, are needed.

## ﻿Taxonomic treatment

### ﻿ Tectaria fungii

Taxon classificationPlantaePolypodialesTectariaceae

S.Y. Dong sp. nov.

1FD17A96-2885-5162-8D00-18247BECAE01

urn:lsid:ipni.org:names:77297479-1

**Figure 6.**
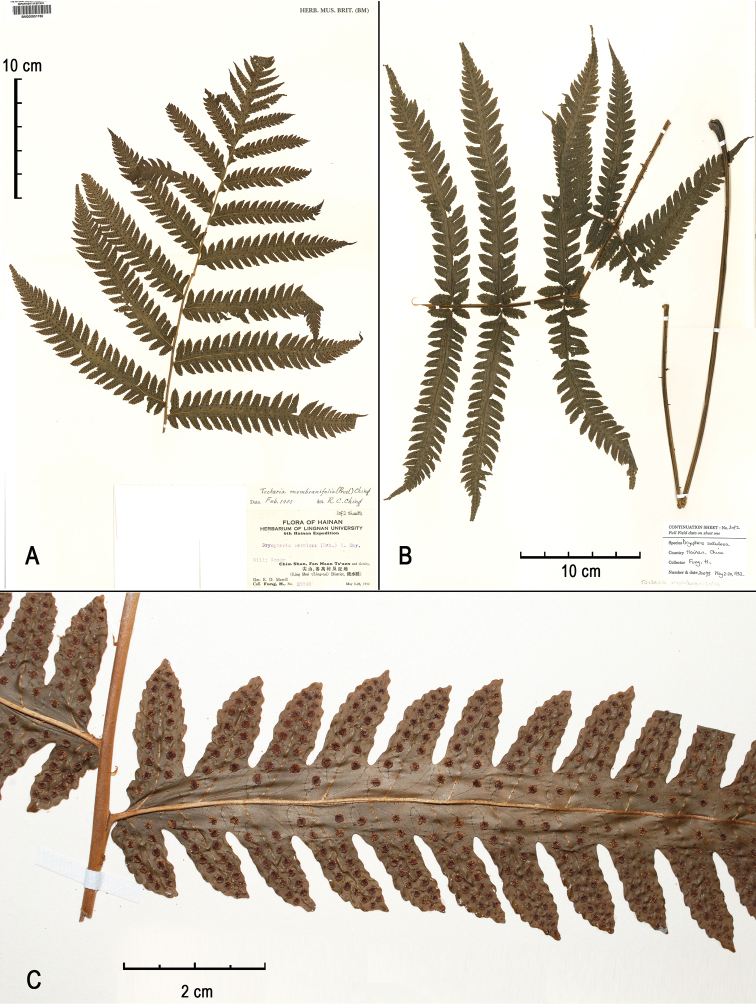
Holotype of *Tectariafungii* (*Fung 20093*, BM), sp. nov. **A** frond’s upper half **B** frond’s lower half **C** details of middle pinnae (abaxial view).

**Figure 7.**
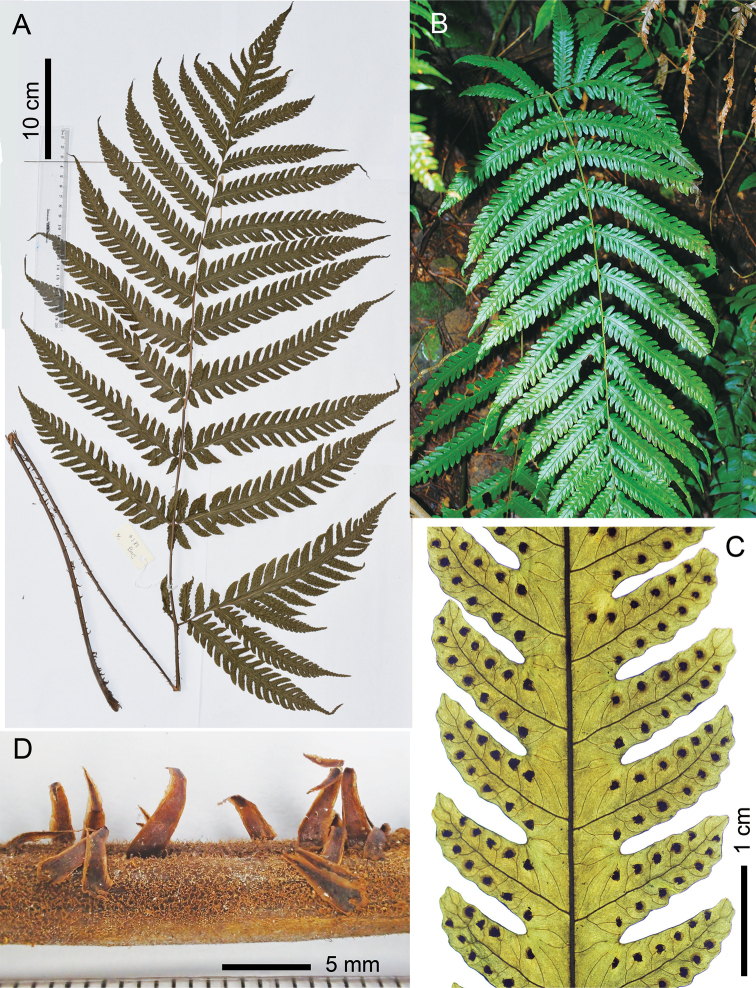
Morphology of *Tectariafungii*, sp. nov. **A** paratype specimen (*Dong 3834*, IBSC), showing outline of a frond **B** habit in the wild **C** portion of a middle pinna, showing veins and sori **D** scales on basal stipe. (All from *Dong 3834*).

Figs 1D , 4A , 6 , 7

#### Type.

**China.** Hainan: Lingshui, 3–20 May 1932, *H. Fung 20093* (holotype, two sheets, BM-000801750!, BM-000801751!; isotypes: E!, K!, US-01580253!, US-01580666!).

#### Diagnosis.

*Tectariafungii* is similar to the sympatric species *T.subsageniacea* and *T.kusukusensis*. It differs from *T.subsageniacea* in its broader (1–1.5 mm versus 0.5–1 mm), brown or obviously bicolour (castaneous with brown margins) (versus black) stipe scales and from *T.kusukusensis* by its nearly hairless laminae (versus frond axes abaxially bearing copious hairs).

#### Description.

**Rhizome** short, erect. **Fronds** slightly dimorphic, rarely obviously dimorphic. **Stipe** stramineous or dark brown, ca. 4 mm in diameter, 50–60 cm long, bearing copious scales towards base and fewer on upper part. **Scales** lanceolate, ca. 6–7 × 1–1.5 mm, brown or castaneous with pale margins. **Lamina** oblong, 55–78 × 30–45 cm, round at base, somewhat suddenly narrowed and acute towards apex, 1-pinnate-pinnatifid, free pinnae (6) 9–12 pairs; basal pinnae triangular, deeply lobed to 1-pinnate at base, (18) 24–26 × 13–18 cm, shortly stalked (0.5–2 cm), with basal basiscopic 2–3 pinnules free and markedly prolonged (up to 16 × 3.5 cm), pinnatifid upwards, acuminate at apex; suprabasal pinnae linear, 16–24 × 3.8–5 cm, sessile or very shortly stalked, deeply lobed 2/3–3/4 of the way to costae, with a pair or only the basal basiscopic lobes almost free; lobes or pinnules anadromous on basal pinnae and catadromous on pinnae above, basal acroscopic lobes slightly prolonged and parallel to rachis, basal basiscopic lobes obliquely spreading, lobes entire (except for those on the base of lower pinnae, which are crenate to pinnatifid), obtuse or acute at apex, sterile lobes larger, usually 2–2.4 × 0.8–0.9 cm, fertile lobes 1.6–1.8 × 0.6 cm. **Veins** free, simple or mostly once forked. **Hairs** short, relative dense on adaxial surface and sparse on abaxial surface of costae; no hairs on abaxial surface between veins; with a few hairs on adaxial surface of lamina, especially on margin and at sinus between lobes. **Sori** terminal on simple veins or on the acroscopic branch of a forked vein, in one row on either side of mid-rib of lobes, medial between mid-rib and margin of lobes. **Indusia** round-reniform, ca. 1 mm in diameter, persistent, usually ciliate at margin.

#### Additional specimens examined.

China. **Hainan**: Baisha, *S.Y. Dong 728* (PE); Baoting, *G.A. Fu 2951* (IBSC); Ledong, *S.Y. Dong 1589* (IBSC); Qiongzhong (Mt. Limushan), *S.Y. Dong 832* (PE); Mt. Wuzhishan, *C. Wang 35347* (IBK, IBSC, PE); *S.Y. Dong 5096* (IBSC), *Wuzhishan Fern Survey 036*, *176*, *& 498* (PE); Mt. Yinggeling, *S.Y. Dong 3834*, *3842*, *3867*, *4430* (IBSC). **Yunnan**: Jinghong, *B.G. Li 98162* (HITBC), *Q.J. Li 42730* (HIBTC); Menghai, *W.M. Chu et al. 15749* (GAUA, PYU), *H. Shang SG2638* (CSH), *X.L. Zhou 5727*, *5731* (CSH); Mengla (Bubeng), *S.Y. Dong 4307*, *4334*, *4825* (IBSC). VIETNAM. **Dak Nong**: Dak Plao, *L. Averyanov et al. 5589* (HN), *5601* (HN, HNU).

#### Distribution and Habitat.

China (Hainan, southern Yunnan) and Vietnam (Dak Nong); terrestrial in montane rainforest, occurring in dense-shady and wet slopes, elev. 600–1300 m, locally common.

#### Etymology.

The specific epithet honours Mr. Hom Fung, who collected lots of plant specimens in Hainan and Guangdong, southern China in 1930s. This species was probably first collected by him from Hainan in 1932.

## Supplementary Material

XML Treatment for Tectaria fungii

## ﻿Appendix 1

List of *Tectaria* samples used for phylogenetic analyses in this study with voucher information (collection number, herbarium and locality) and GenBank numbers for *rbcL*, *atpB*, *rps16–matK* plus *matK*, *ndhF* plus *ndhF-trnN* and *trnL-F*. GenBank numbers for newly-generated sequences are in bold and a dash indicates data absent.

﻿***Tectariaaenigma*** (Copel.) C.W. Chen & C.J. Rothf., *DNK 575* (UC), Philippines, KY927533, –, KJ196548, –, KY927538. *Tectariaaurita* (Sw.) S. Chandra, *Dong 3386* (IBSC), Indonesia (Java), KJ196849, KJ196404, KJ196548, KJ196762, KJ196631. *Tectariaaustrosinensis* (Christ) C. Chr., *Dong 3602* (IBSC), China (Yunnan), KJ196899, KJ196447, KJ196516, KJ196804, KF561670; *Dong 3617* (IBSC), China (Yunnan), KJ196847, KJ196446, KJ196517, KJ196803, KJ196629. *Tectariaborneensis* S.Y. Dong, *Dong 3438* (IBSC), Indonesia (Java), KJ196854, KJ196489, KJ196514, KJ196767, KJ196642. *Tectariachinensis* (Ching & Chu H. Wang) Christenh., *Dong 4291* (IBSC), China (Yunnan), MH542574, MH542584, MH542595, MH542606, MH542618; *Dong 4343* (IBSC), China (Yunnan), MF623757, MF623685, MF623709, MF623733, MF623780; *Dong 4855* (IBSC), Vietnam, MH542575, MH542585, MH542596, MH542607, MH542619. *Tectariadissecta* (G. Forst.) Lellinger, *CYH 20140503014* (IBSC, TAIF), China (Taiwan), MH542570, MH542580, MH542591, MH542603, MH542614; *Dong 4101* (IBSC), Philippines (Luzon), AWD73648, AWD73600, –, AWD73624, –. *Tectariafungii* S.Y. Dong (sp. nov.), *Dong 3834* (IBSC), China (Hainan), KJ196826, KJ196505, KJ196591, KJ196751, KJ196703; *Dong 4334* (IBSC), China (Yunnan), MH542571, MH542581, MH542592, MH542604, MH542615; *Dong 4430* (IBSC), China (Hainan), W795604, –, MW795617, MW795625, –. *Tectariafuscipes* (Wall. ex Bedd.) C. Chr., *CYH 20140322019* (IBSC, TAIF), China (Taiwan), MH542568, MH542578, MH542589, MH542601, MH542612; *CYH 20140503020* (TAIF), China (Taiwan), MH542569, MH542579, MH542590, MH542602, MH542613; *Dong 4271* (IBSC), China (Yunnan), OL828756, OL828758, NA, OL963688, OL828760; *Dong 5187* (IBSC), Bangladesh, –, –, –, MW795618, MW795626; *Dong 5194* (IBSC), Bangladesh, MW795597, MW795605, MW795611, MW795619, MW795627; *Knapp 3198* (P), China (Taiwan), KY937334, –, KY937227, –, KY937497. *Tectariagigantea* (Blume) Copel., *Dong 3380* (IBSC), Indonesia (Java), KJ196853, KJ196403, KJ196530, KJ196737, KJ196660. *Tectariagymnocarpa* Copel., *Dong 3967* (IBSC), Papua New Guinea (Kimbe), MF623765, MF623693, MF623717, MF623741, MF623786. *Tectariaheterocarpa* (Bedd.) C.V. Morton, *Dong 5189* (IBSC), Bangladesh, MW795598, MW795606, MW795612, MW795620, MW795628. *Tectariaingens* (Atk. ex C.B. Clarke) Holttum, *Zuo 4218* (KUN), China (Yunnan), MW795599, MW795607, MW795613, MW795621, MW795629. *Tectariakusukusensis* (Hayata) Lellinger, *CYH 20111228044* (IBSC, TAIF), China (Taiwan), MF623770, MF623698, MF623722, MF623746, MF623790; *Knapp 3336* (IBSC), China (Taiwan), MH542573, MH542583, MH542594, –, MH542617; *Zhang 6466* (CDBI, MO, VNMN), Vietnam, KP271079, –, KU605135, –, KP271096. *Tectariamulticaudata* (C.B. Clarke) Ching, *CYH 20080816006* (IBSC, TAIF), China (Taiwan), MH542572, MH542582, MH542593, MH542605, MH542616; *Wade 1382* (IBSC), Vietnam, KJ196834, KJ196425, KJ196558, KJ196756, KJ196713. *Tectarianayarii* Mazumdar, *Wade s.n.* (TAIF), Philippines (Luzon), KJ196823, KJ196405, KJ196594, KJ196722, KJ196699. *Tectariaparadoxa* (Fée) Sledge, *Chiou 15285* (TAIF), India, MW795600, –, –, –, MW795630. *Tectariapolymorpha* (Wall. ex Hook.) Copel., *Dong 3554* (IBSC), China (Yunnan), KJ196889, KJ196477, KJ196524, KJ196794, KJ196657. *Tectariaprofereoides* (Christ) S.Y. Dong, *Dong 4939* (IBSC), Malaysia, MW795601, MW795608, MW795614, MW795622, MW795631; *Dong 4961* (IBSC), Malaysia, MW795602, MW795609, MW795615, MW795623, MW795632. *Tectariapsomiocarpa* S.Y. Dong, *Chen s.n.* (IBSC), Philippines (Luzon), KJ196822, KJ196502, KJ196595, KJ196723, KJ196698. *Tectariasabahensis* C.W. Chen & C.J. Rothf., *Wade 4705* (TAIF), Malaysia, KY927534, –, KY927535, –, KY927537. *Tectariasagenioides* (Mett.) Christenh., *Dong 1599* (IBSC), China (Hainan), KJ196896, KJ196436, KJ196550, KJ196760, KJ196625; *Dong 3603* (IBSC), China (Yunnan), KJ196897, KJ196437, KJ196518, KJ196801, KF561672. *Tectariasetulosa* (Baker) Holttum, *Dong 3049* (IBSC), China (Guangdong), –, KJ196428, KJ196527, KJ196791, KJ196670; *Jin et al. 282* (IBSC, PE), China (Yunnan), –, KJ196427, KJ196557, KJ196757, KJ196714. *Tectariasubfuscipes* (Tagawa) C.M. Kuo, *CYH 20140503023* (IBSC, TAIF), China (Taiwan), MH542566, –, MH542587, MH542599, MH542610; *Deng D20140518002* (IBSC, TAIF), China (Taiwan), MH542567, MH542577, MH542588, MH542600, MH542611; *Knapp 3199* (P), China (Taiwan), KY937373, –, KY937273, –, KY937563. *Tectariasubglabra* (Holttum) S.Y. Dong, *Dong 3450* (IBSC), Indonesia (Java), KJ196807, KJ196406, KJ196532, KJ196738, KJ196676. *Tectariasubsageniacea* (Christ) Christenh., *Chao 1553* (IBSC), Vietnam, KJ196900, KJ196414, KJ196560, KJ196733, KJ196711; *Dong 3557* (IBSC), China (Yunnan), KJ196880, KJ196415, KJ196529, KJ196782, KJ196665; *Dong 3856* (IBSC), China (Hainan), MF623767, MF623695, MF623719, MF623743, MF623788; *Dong 4585* (IBSC), Vietnam, MH542564, MH542576, –, MH542597, MH542608; *Dong 4587* (IBSC), Vietnam, MH542565, –, MH542586, MH542598, MH542609. Tectariacf.subfuscipes, *Fraser-Jenkins FN153* (TAIF), India (Meghalaya), OL828757, NA, OL828759, OL963689, OL828761; *Knapp 3200* (P), China (Taiwan), KY937374, –, –, –, KY937564; *Tectaria* sp., *Wade 4087* (IBSC), Solomon Islands, MW795603, MW795610, MW795616, MW795624, MW795633.
